# Supplementary material for: Causal Relationship between Aging and Anorexia Nervosa: A White-Matter-Microstructure-Mediated Mendelian Randomization Analysis
Source: Biomedicines. 2024 Aug 16;12(8):1874. doi: 10.3390/biomedicines12081874 (PMC11351342; doi:10.3390/biomedicines12081874)
Supplement: Supplementary file 1 [file biomedicines-12-01874-s001.zip › Supplementary Figure/Figure S4/Singe SNP plot of MR analysis for TL to ubm-b-1887.pdf]

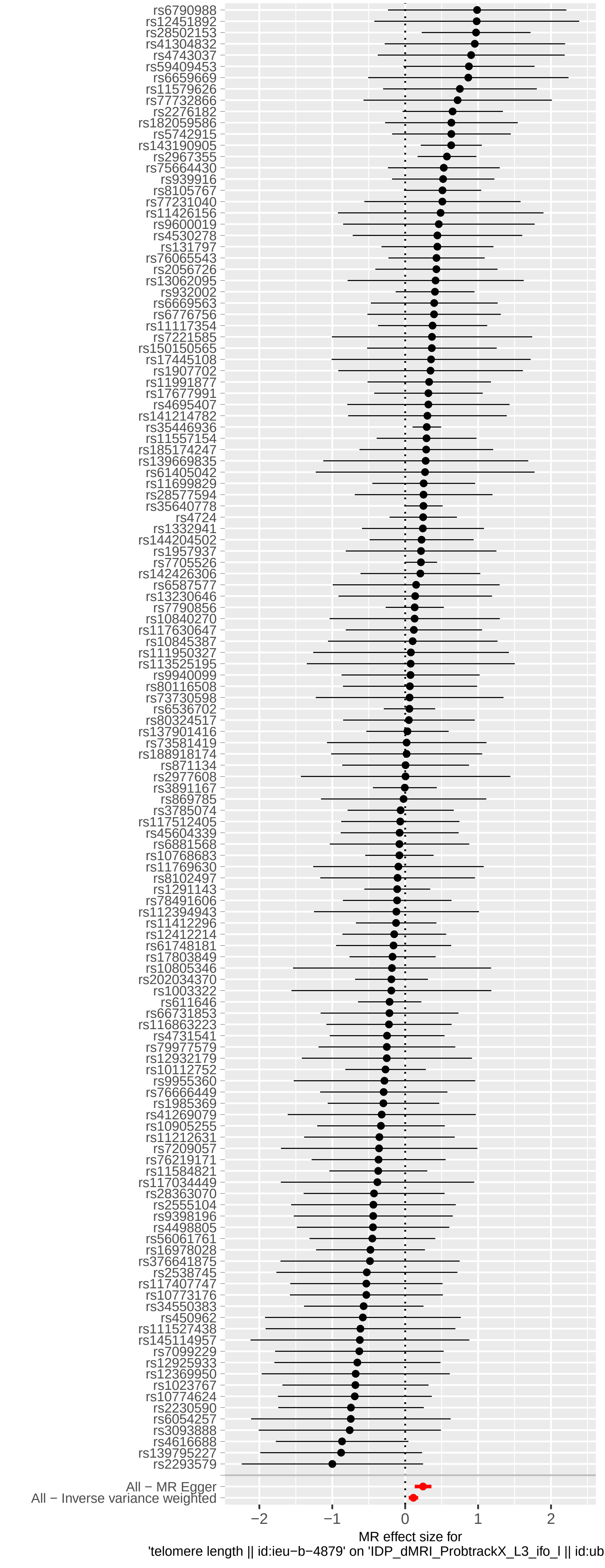
rs6790988
rs12451892
rs28502153
rs41304832
rs4743037
rs59409453
rs6659669
rs11579626
rs77732866
rs2276182
rs182059586
rs5742915
rs143190905
rs2967355
rs75664430
rs939916
rs8105767
rs77231040
rs11426156
rs9600019
rs4530278
rs131797
rs76065543
rs2056726
rs13062095
rs932002
rs6669563
rs6776756
rs11117354
rs7221585
rs150150565
rs17445108
rs1907702
rs11991877
rs17677991
rs4695407
rs141214782
rs35446936
rs11557154
rs185174247
rs139669835
rs61405042
rs11699829
rs28577594
rs35640778
rs4724
rs1332941
rs144204502
rs1957937
rs7705526
rs142426306
rs6587577
rs13230646
rs7790856
rs10840270
rs117630647
rs10845387
rs111950327
rs113525195
rs9940099
rs80116508
rs73730598
rs6536702
rs80324517
rs137901416
rs73581419
rs188918174
rs871134
rs2977608
rs3891167
rs869785
rs3785074
rs117512405
rs45604339
rs6881568
rs10768683
rs11769630
rs8102497
rs1291143
rs78491606
rs112394943
rs11412296
rs12412214
rs61748181
rs17803849
rs10805346
rs202034370
rs1003322
rs611646
rs66731853
rs116863223
rs4731541
rs79977579
rs12932179
rs10112752
rs9955360
rs76666449
rs1985369
rs41269079
rs10905255
rs11212631
rs7209057
rs76219171
rs11584821
rs117034449
rs28363070
rs2555104
rs9398196
rs4498805
rs56061761
rs16978028
rs376641875
rs2538745
rs117407747
rs10773176
rs34550383
rs450962
rs111527438
rs145114957
rs7099229
rs12925933
rs12369950
rs1023767
rs10774624
rs2230590
rs6054257
rs3093888
rs4616688
rs139795227
rs2293579
All − MR Egger
All − Inverse variance weighted
−2
−1
0
1
2
MR effect size for
'telomere length || id:ieu−b−4879' on 'IDP_dMRI_ProbtrackX_L3_ifo_l || id:ub
